# Delivery of xenon-containing echogenic liposomes inhibits early brain injury following subarachnoid hemorrhage

**DOI:** 10.1038/s41598-017-18914-6

**Published:** 2018-01-11

**Authors:** Yi-Feng Miao, Tao Peng, Melanie R. Moody, Melvin E. Klegerman, Jaroslaw Aronowski, James Grotta, David D. McPherson, Hyunggun Kim, Shao-Ling Huang

**Affiliations:** 1 Division of Cardiovascular Medicine, Department of Internal Medicine, The University of Texas Health Science Center at Houston, Houston, TX 77030 USA; 2 Department of Neurology, The University of Texas Health Science Center at Houston, Houston, TX 77030 USA; 3 Stroke Program, Memorial Hermann Hospital, Houston, TX 77030 USA; 4 Department of Biomechatronic Engineering, Sungkyunkwan University, Suwon, Gyeonggi 16419 Korea

## Abstract

Xenon (Xe), a noble gas, has promising neuroprotective properties with no proven adverse side-effects. We evaluated neuroprotective effects of Xe delivered by Xe-containing echogenic liposomes (Xe-ELIP) via ultrasound-controlled cerebral drug release on early brain injury following subarachnoid hemorrhage (SAH). The Xe-ELIP structure was evaluated by ultrasound imaging, electron microscopy and gas chromatography-mass spectroscopy. Animals were randomly divided into five groups: Sham, SAH, SAH treated with Xe-ELIP, empty ELIP, or Xe-saturated saline. Treatments were administrated intravenously in combination with ultrasound application over the common carotid artery to trigger Xe release from circulating Xe-ELIP. Hematoma development was graded by SAH scaling and quantitated by a colorimetric method. Neurological evaluation and motor behavioral tests were conducted for three days following SAH injury. Ultrasound imaging and electron microscopy demonstrated that Xe-ELIP have a unique two-compartment structure, which allows a two-stage Xe release profile. Xe-ELIP treatment effectively reduced bleeding, improved general neurological function, and alleviated motor function damage in association with reduced apoptotic neuronal death and decreased mortality. Xe-ELIP alleviated early SAH brain injury by inhibiting neuronal death and bleeding. This novel approach provides a noninvasive strategy of therapeutic gas delivery for SAH treatment.

## Introduction

Subarachnoid hemorrhage (SAH) is a medical emergency with high mortality. Epidemiologic studies have shown that the overall mortality rates range from 32–67%, with 10–20% of these patients dying before reaching hospital, 33% dying within 48 hours, and 40–60% dying within 30 days following SAH onset^1^. Thirty percent of survivors suffer various degrees of morbidity. Vasospasm is considered the primary pathological event following rupture and bleeding^2,3^. A recent multicenter clinical trial indicates that prevention of delayed vasospasm does not improve mortality and morbility in SAH patients^4,5^. This finding has focused the role of stabilization of early brain injury resulting from SAH. Substantial evidence indicates that early brain injury begins minutes after aneurysmal rupture and plays an important role in the patients’ subsequent condition, including overall mortality and delayed ischemic injury^6,7^. Therefore, effective early neuroprotective treatment has become a major focus of SAH management^7^.

A number of putative neuroprotective therapeutics showing efficacy in animal models have failed in clinical trials^8^. Principle reasons include marginal efficacy with severe systemic adverse effects^9^, insufficient local drug concentration due to inefficient drug delivery methods^10^, or delay in onset of drug action following stroke^11^. If a neuroprotective agent providing high diffusion into the hypoperfused brain with low systemic side effects in combination with a novel therapeutic delivery method for enhanced local release could be developed, it would have great potential for early treatment of SAH at the time of first response prior to neurological evaluation and treatment in the hospital setting.

Xenon (Xe), a noble gas having antiproteolytic properties^12^, is a well-known neuroprotectant in treatment of brain injury^13–17^. Unlike other therapeutics, Xe is a small molecule that can rapidly diffuse across the blood-brain barrier (BBB) and transit across cell membranes with no proven adverse side effects^18,19^. Pharmacologically, Xe provides concentration-dependent protection against neuronal injury by blocking excitotoxicity^19^. Xe protects against oxygen and glucose deprivation (OGD) as well as against hypoxia/ischemia by alteration of molecules involved in neuronal ischemic tolerance^20^. Xe helps induce transcription of several pro-survival genes, including brain-derived neurotrophic factor (BDNF) and pro-survival proteins such as Bcl_2_, which promotes cell tolerance to brain injury^20,21^.

Current methodologies for Xe delivery primarily involve inhalation. Inhalation of Xe is expensive and involves complicated medical instruments, and availability of Xe in large quantities is not feasible. This results in the difficulty of developing a continuous Xe inhalation strategy for patients in the field. Carriers containing gas molecules may provide a tool to overcome these limitations. Liposomes (lipid vehicles) have hydrophilic and hydrophobic compartments, which are ideal for encapsulation of payloads having different biochemical properties^22,23^. We have developed Xe-containing echogenic liposomes (Xe-ELIP) using a unique freeze-under-pressure method to entrap Xe molecules into liposomes^24^. Our ELIP can entrap air and other gases for enhanced contrast in ultrasound imaging. Currently, several other lipid-based contrast agents are commercially available for patient use^25,26^. In addition to being used as an ultrasound contrast agent, our ELIP can serve as an ultrasound-responsive carrier for Xe delivery. We have demonstrated the neuroprotective effect of Xe-ELIP in ischemic stroke following intravenous administration of Xe-ELIP in association with ultrasound application over the carotid arteries to trigger Xe release from Xe-ELIP^15^.

For the purpose of stroke treatment, it is not possible to determine which type of stroke (ischemic or hemorrhagic) a patient is having in the field when paramedics initially evaluate the patient. If Xe-ELIP can provide neuroprotective effects on the brain in patients with both stroke types, it would greatly help by extending the time window prior to conventional stroke treatment, i.e., before hospital evaluation of stroke type by computed tomography (CT). In this study, we investigated the neuroprotective effects of Xe-ELIP delivery in treatment of early brain injury following SAH.

## Results

### Xe Gas Incorporation in Xe-ELIP

**Figure 1:**
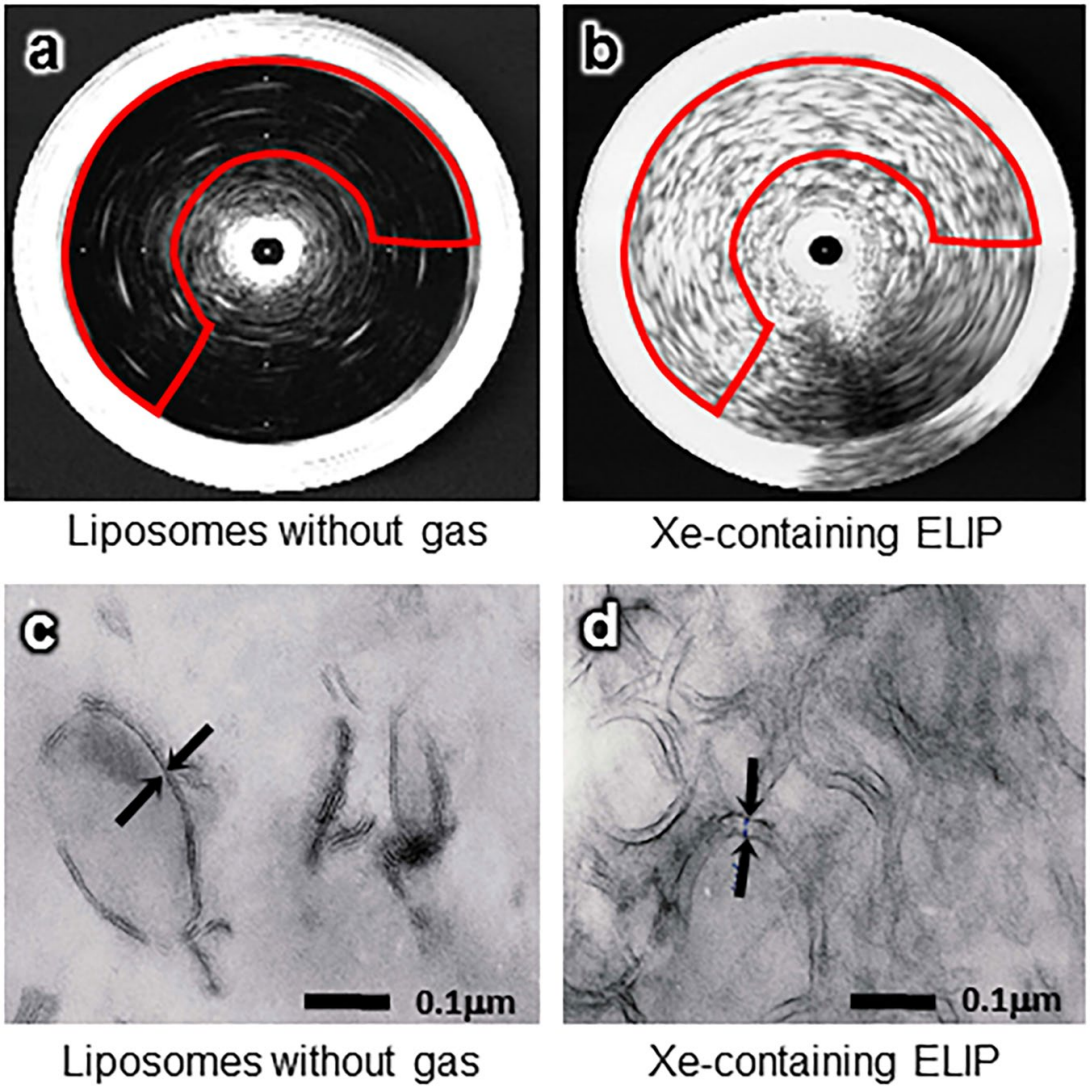
Imaging of liposomes without gas loading and Xe-ELIP using IVUS and electron microscopy. (**a**) IVUS image of normal liposomes (without gas inside), (**b**) Xe-containing ELIP (white contents in the marked area indicate ultrasound reflectivity from Xe gas bubbles in Xe-ELIP), (**c**) Electron microscopic image of normal liposomes (without gas inside), and (**d**) gas-containing liposomes (gas-containing liposomes have wider lipid bilayers). Red lines refer to the region of interest where acoustic reflectivity was measured. Arrows indicate the thickness of the lipid bilayer.

Gas chromatography-mass spectroscopy (GC-MS) demonstrated that our encapsulation method allowed 20 μl Xe to be incorporated per mg lipids. Little acoustic reflectivity was found in traditional liposomes (Fig. 1a), while high acoustic reflectivity was shown in Xe-containing ELIP (Fig. 1b) demonstrating the existence of Xe bubbles in Xe-ELIP. We measured acoustic reflectivity in the same region of interest (delineated in red lines) in both images, excluding the white area near the center which involves an artificial reflection from the IVUS catheter (the black circle area in the center). After the bubbles lost their reflectivity following ultrasound exposure, GC-MS indicated that 34% of Xe still remained in the Xe-ELIP (not enough to have an ultrasound-controlled effect, but still enough to have resided Xe release). Investigation by electronic microscopy demonstrated that the width of the lipid bilayer of gas-loaded ELIP (Fig. 1d) was wider than in liposomes without gas entrapment (Fig. 1c).

### ELIP Adhesion to Damaged Endothelial Cells

**Figure 2:**
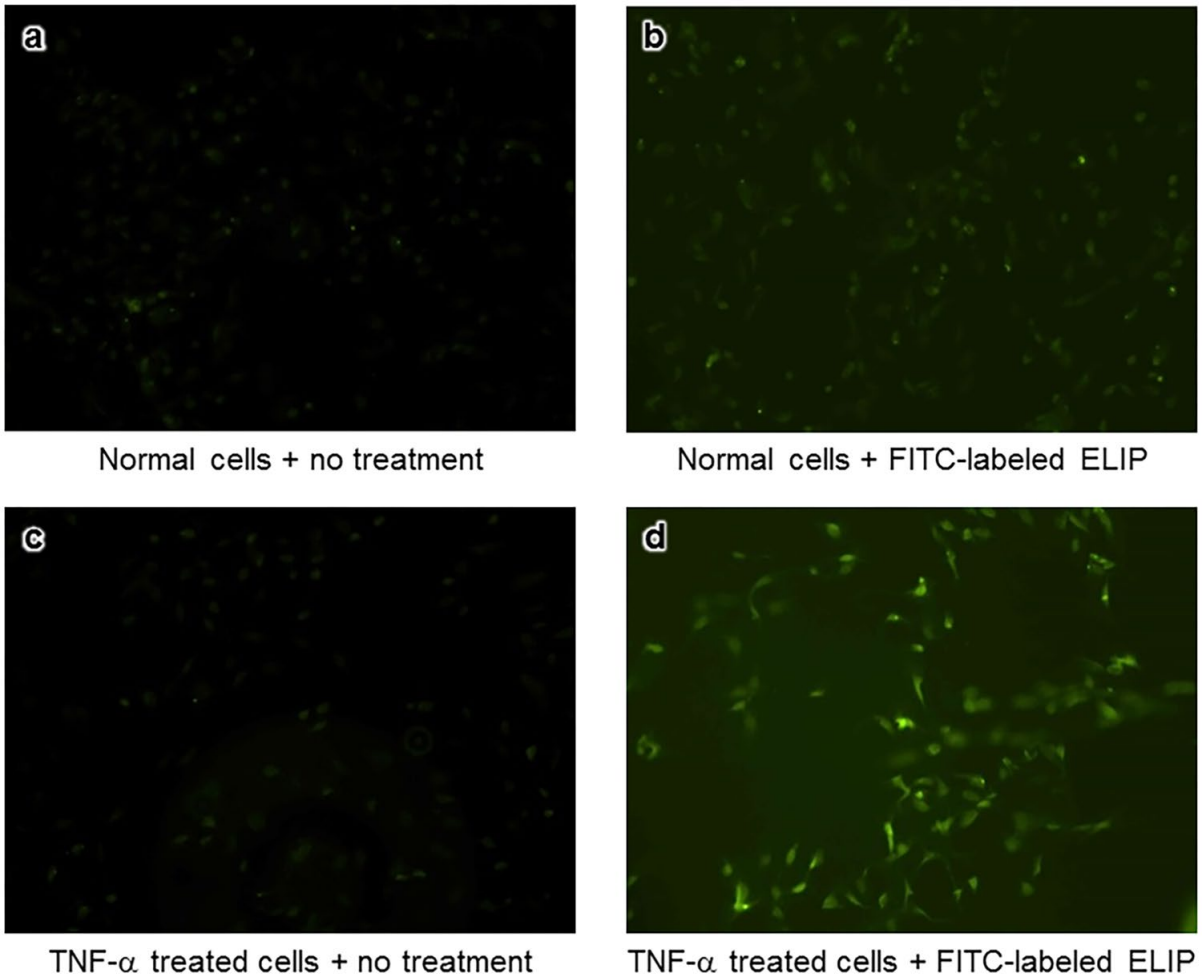
Fluorescence microscopy of (**a**) normal endothelial cells without any treatment, (**b**) normal endothelial cells incubated with FITC-labeled ELIP, (**c**) TNF-α treated cells without any treatment, and (**d**) TNF-α treated cells incubated with FITC-labeled ELIP.

FITC-ELIP adhesion to damaged cells was observed under fluorescence microscopy. Normal and TNF-α treated endothelial cells did not demonstrate fluorescence (Fig. 2a,c). Weak fluorescence was found in normal endothelial cells incubated with FITC-labeled ELIP. Strong fluorescence was found in the TNF-α treated cells incubated with FITC-labeled ELIP (Fig. 2d), indicating that these ELIP adhered to damaged endothelial cells.

### Evaluation of Neurological and Motor Function

**Figure 3:**
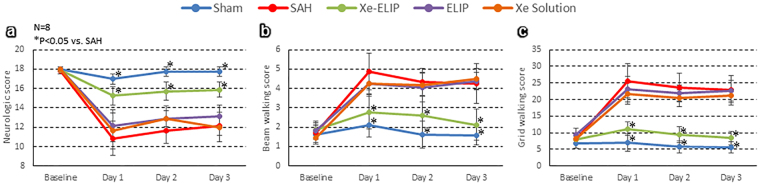
Effect of Xe-ELIP treatment. (**a**) general neurological and motor function, (**b**) beam walking, and (**c**) grid walking on day 1, day 2 and day 3 after SAH onset.

An 18-point scoring test was used for overall neurological evaluation (Table 1). Figure 3 demonstrates that Xe-ELIP improved SAH induced neurological disfunction (Fig. 3a, P < 0.01) from the first day to the third day after SAH onset. Beam walking (Fig. 3b) and grid walking (Fig. 3c) tests revealed that Xe-ELIP improved motor function starting also from the first day.

**Table 1:** Post-SAH neurological evaluation (18 scores)^54^.

**Test**	**Score**			
	**0**	**1**	**2**	**3**
Spontaneous Activity (in cage for 5 min)	No movement	Barely moves position	Moves but does not approach at least three sides of cage	Moves and approaches at least three sides of cage
Spontaneous movements of all limbs	No movement	Slight movement of limbs	Moves all limbs but slowly	Moves all limbs same as pre-SAH
Movements of forelimbs (outstretching while held by tail)	No outstretching	Slight outstretching	Outstretching is limited and less than pre-SAH	Outstretching is same as pre-SAH
Climbing wall of wire cage		Fails to climb	Climbs weakly	Normal climbing
Reaction to touch on both side of trunk		No response	Weak response	Normal response
Response to vibrissae touch		No response	Weak response	Normal response

### Decrease of Hematoma

**Figure 4:**
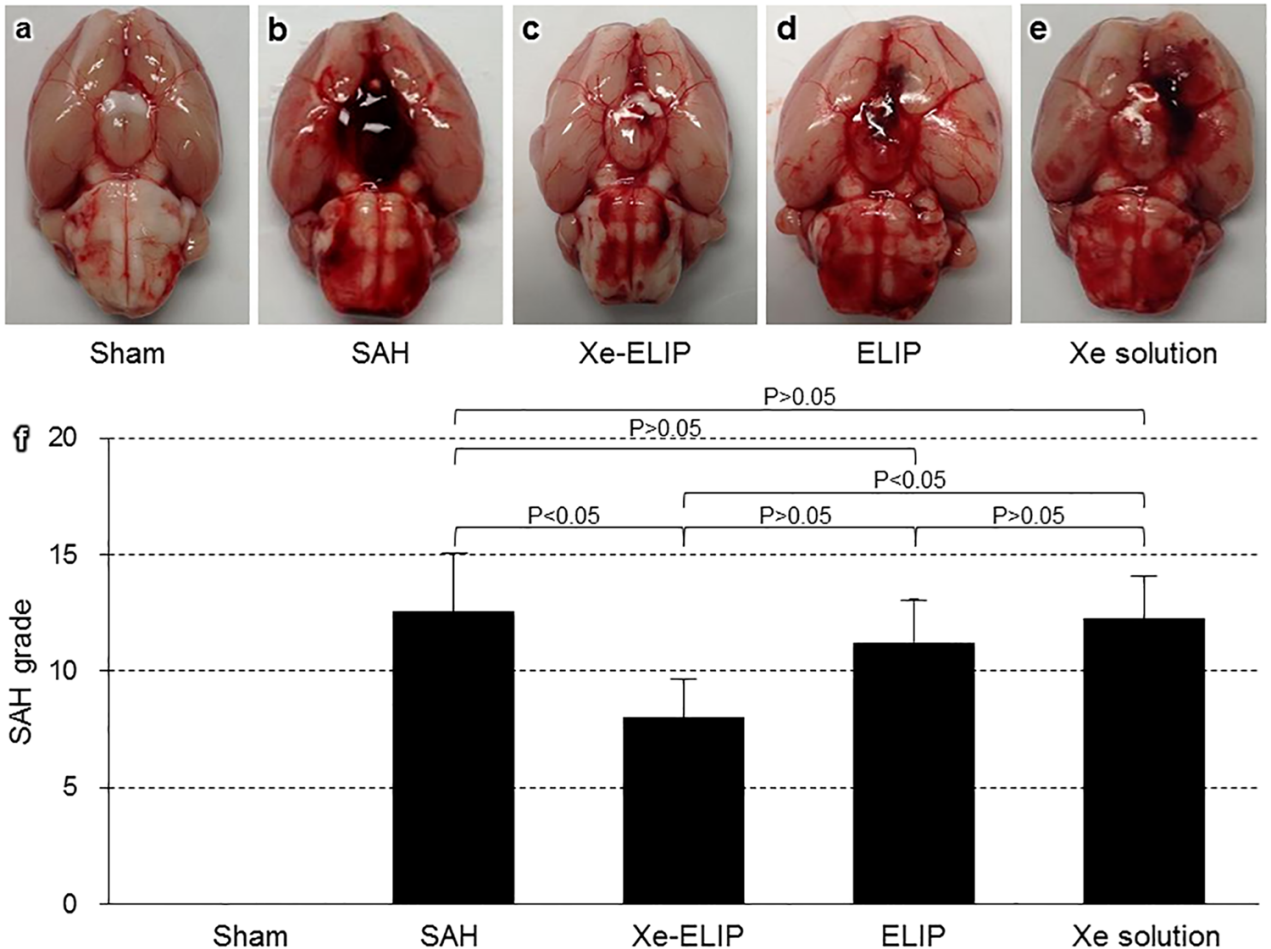
Effect of Xe-ELIP treatment on brain hemorrhage. Gross anatomy of brain hemorrhage in (**a**) Sham, (**b**) SAH without treatment, (**c**) Xe-ELIP, (**d**) ELIP, and (**e**) Xe solution groups. (**f**) Grading of the severity of SAH grade was determined as the sum of the scores from the 6 subsections. Data are shown as mean ± SEM (n = 8 animals/group).

**Figure 5:**
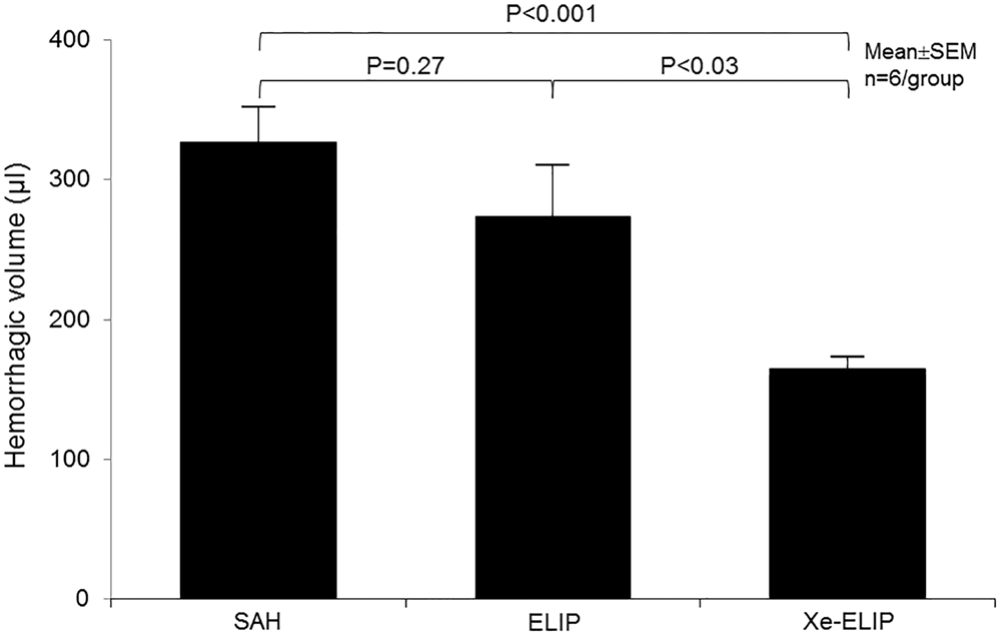
Quantitation of hemorrhagic volume in the Sham, SAH, and Xe-ELIP groups. Hemorrhagic volume was measured using a spectrophotometer. Data are shown as mean ± SEM (n = 6 animals/group).

The effect of Xe-ELIP on reducing hematoma in the SAH brain was quantitatively evaluated using SAH grade (Fig. 4) and spectrophotometric assay (Fig. 5).

SAH scores were 0 in the Sham group, 12.6 ± 2.50 in the SAH group, 8.0 ± 1.63 in the Xe-ELIP group, 11.3 ± 1.83 in the ELIP group, and 12.3 ± 1.83 in the Xenon group, respectively. Xe-ELIP with ultrasound treatment lessened the degree of hemorrhage compared with the SAH group (P < 0.05). Neither ELIP nor Xe solution reduced the degree of hemorrhage compared to the SAH group (Fig. 4).

At 2 hours after surgery, spectrophotometric assay was performed to measure hemorrhagic volume. The hemorrhagic volumes were 327 ± 25 μl in the SAH group, 274 ± 37 μl in the ELIP group (P = 0.27 vs. no treatment), 165 ± 9 μl in the Xe-ELIP group (P < 0.001 vs. no treatment; P = 0.026 vs. ELIP treatment). Xe-ELIP treatment markedly reduced hemorrhagic volume compared to other groups (Fig. 5).

### Neuronal Cell Death

**Figure 6:**
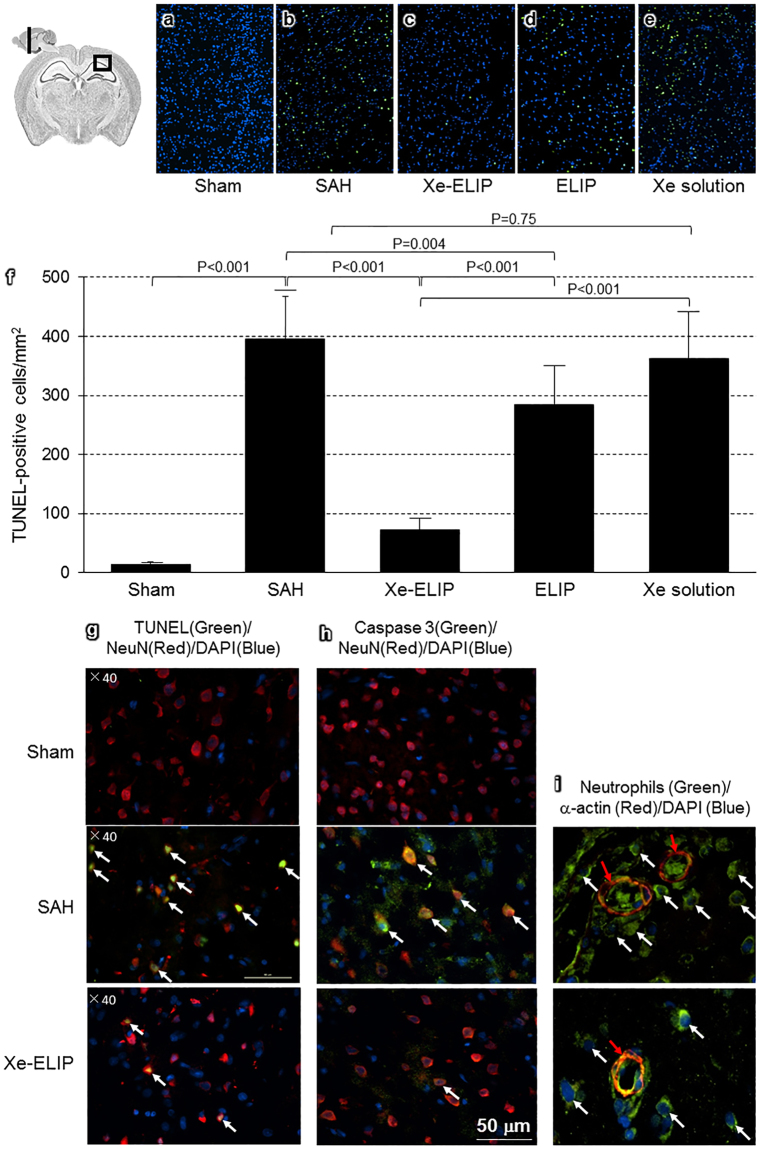
Immunofluorescent staining of the coronal sections of brain subcortex. TUNEL staining in (**a**) Sham, (**b**) SAH, (**c**) Xe-ELIP, (**d**) ELIP, and (**e**) Xe solution groups. The TUNEL-positive cells were stained with green fluorescence. Nuclei were counter stained with DAPI (blue). (**f**) The number of TUNEL-positive cells was calculated per mm2. Data are shown as mean ± SEM (n = 8 animals/group). (**g**) Colocalization of the TUNEL-positive cells (green) with the neurons (red). Arrows indicate colocalized TUNEL-positive cells. (**h**) Colocalization of the caspase 3-positive cells (green) with the neurons (red). Arrows indicate colocalized caspase 3-positive cells. (**i**) Colocalization of neutrophils (green) with blood vessel (red) in the subcortex in the SAH and Xe-ELIP groups. Arrows indicate neutrophil infiltration in the brain tissue.

TUNEL staining was performed to examine the effect of Xe-ELIP on neuronal cell death at 72 hours after SAH onset. The mean number of TUNEL-positive cells in the subcortex area in a microscopic field at a 10 × objective was recorded and compared (Fig. 6). In the Sham group, TUNEL-positive cells were 14 ± 1 (Fig. 6a). SAH induced an increase in the incidence of TUNEL-positive cells (396 ± 22, P < 0.001 vs. Sham, Fig. 6b). Xe-ELIP treatment significantly reduced the number of TUNEL-positive cells to 72 ± 9 (P < 0.01 vs. SAH, Fig. 6c). ELIP only treatment slightly reduced TUNEL-positive cells with no difference to SAH (284 ± 48, P = 0.96 vs. SAH, Fig. 6d). Xe-solution treatment did not show a therapeutic effect on neuronal cell death (361 ± 65, P = 0.25 vs. SAH, Fig. 6e). This indicates that intravenous Xe-ELIP administration clearly inhibited hemorrhage-induced neuronal cell death.

### Colocalization of Immunoreactivity

We evaluated TUNEL-positive cells and caspase 3 expression to assess colocalization with neurons. At 72 hours after SAH, the TUNEL-positive cells (Fig. 6g) and the caspase 3 expression (Fig. 6h) increased, demonstrating colocalization with the neuronal cells, increasing with SAH and decreasing following Xe-ELIP treatment.

### Three-Day Mortality

In this study, the SAH model was induced by endovascular perforation of the middle cerebral artery (MCA) to mimic clinical SAH^27^. No animal (0%) died in the Sham group (n = 8). Eight animals (36.4%) died in the SAH group (n = 22); 4 animals (20%) in the Xe-ELIP treatment group (n = 20); 2 animals (20%) in the ELIP only group (n = 10); and 3 animals (27.3%) in the Xe solution group (n = 11). Most (80%) deaths occurred on the first day after SAH onset. There was no difference in body weight changes of the animals over 3 days following surgery. The animals in the SAH and the Xe-ELIP groups lost 29 ± 8% and 26 ± 4% of their body weight, respectively.

## Discussion

We have demonstrated that Xe-ELIP can deliver a sufficient amount of Xe into the cerebral circulation and alleviate early SAH brain injury, resulting in reduced mortality and morbidity at day 3. Our previous data have demonstrated that Xe is released from Xe-ELIP in a biphasic manner^15^. Here, we discovered that the biphasic release manner is related to its structure. Ultrasound imaging and electron microscopy have demonstrated that our Xe-ELIP has a unique two-compartment structure, which allows a two-stage Xe release profile: dissolved Xe atoms existing in the lipid bilayer (resulting in a wider lipid bilayer) and Xe bubbles either within the bilayer or in the internal aqueous compartment, which accounts for the ultrasound reflectivity, (i.e., echogenicity). Xe solubility is 0.097 ml/ml in water and 1.85 ml/ml in lipid^28^. When dissolved in the lipid, Xe molecules partition into the lipid bilayer leading to an increased lipid shell thickness. Each 16-carbon fatty acid chain lipid can associate with more than three Xe atoms^29,30^ by hydrophobic forces. This two-compartment structure of Xe-ELIP provides different characteristics of Xe release: Xe bubbles respond to ultrasound-triggered release from Xe-ELIP. while dissolved Xe releases slowly from lipid bilayer. We previously found that 62 ± 19% of Xe content was released following ultrasound activation, and sustained Xe release lasted for up to 10 hours^15^. This is confirmed by our GC-MS data showing that 34% of Xe still remains after ultrasound exposure. This remaining Xe can release slowly from the lipid bilayer for a longer duration of effect. These controlled release characteristics of Xe-ELIP are important when delivering sufficient therapeutics to the target tissue.

**Figure 7:**
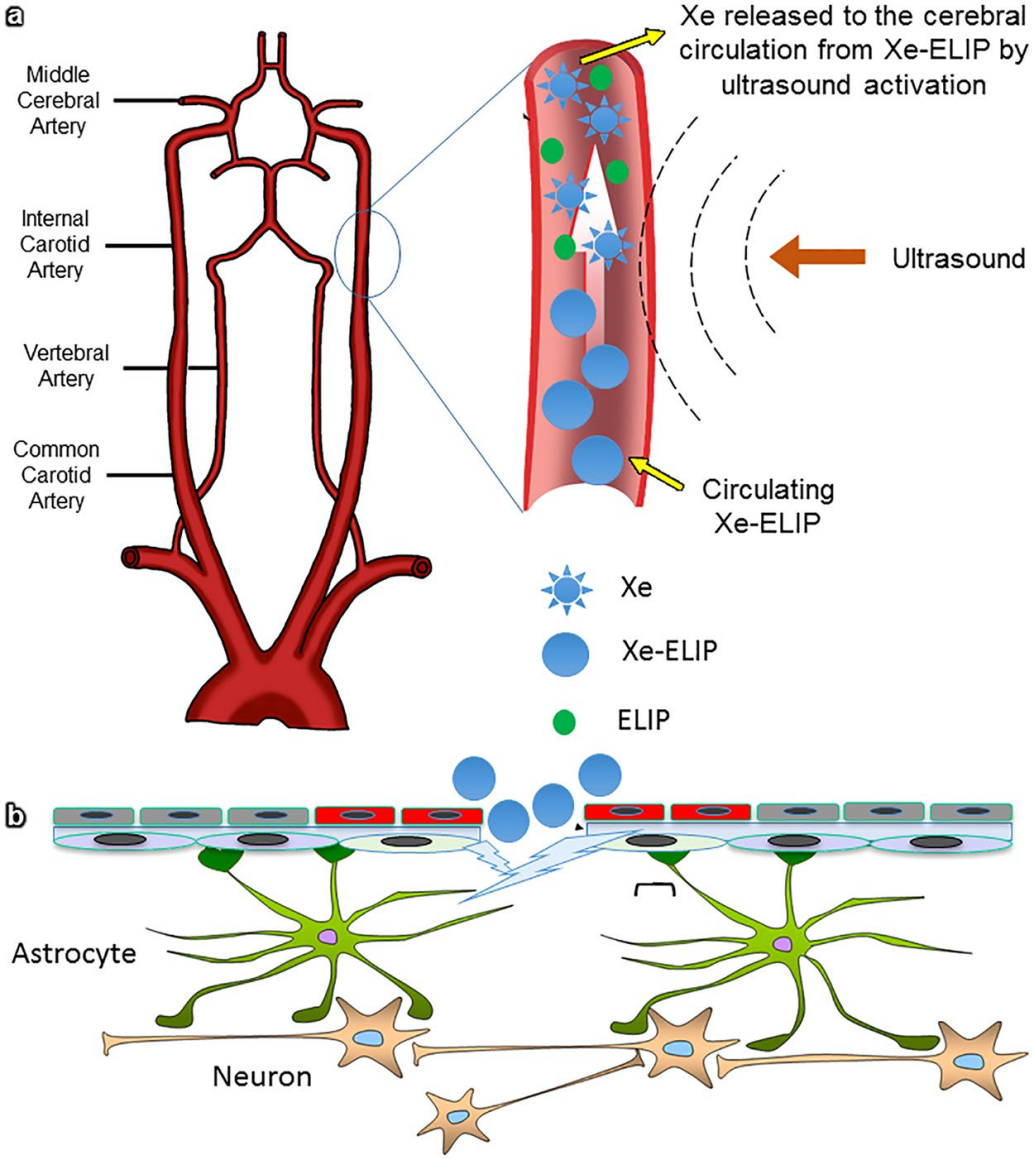
(**a**) Schematic of the Xe delivery strategy using Xe-ELIP. Xe-ELIP can be infused intravenously and ultrasound applied over the common carotid artery for ultrasound-triggered priminary Xe release from circulating Xe-ELIP into the brain. (**b**) Xe-ELIP adhesion to the damaged endothelial cells to patch the hole and localize secondary release. Normal endothelium is negatively charged as the endothelium itself has an inner glycoprotein coat of negatively charged particles. After cellular damage, collagen and tissue underneath the endothelium are exposed and have positive charges. Xe-ELIP have slight negative charges and stick to the damaged vascular site to aid in repair and allow localized Xe release.

Anatomically, there are four major arteries passing through the neck toward the brain – two internal carotid arteries (ICA) and two vertebral arteries. Physiologically, the ICA and vertebral arteries supply approximately 80% and 20% of blood to the brain, respectively. Ultrasound activation on the neck over the ICA can specifically control therapeutic release into the brain (Fig. 7a). In previous studies, we demonstrated that ultrasound-trrigered Xe release from circulating Xe-ELIP into the cerebral circulation provided enhanced local therapeutic effect^15,31^. Our *in-vitro* studies using cultured endothelial cells demonstrated that considerably more Xe-ELIP adhered to damaged endothelial cells compared to normal endothelial cells (Fig. 2). As Xe-ELIP pass through the MCA where the perforation occurs, adhesion of these Xe-ELIP to the damaged neurovascular endothelium has two effects (Fig. 7b) – repair of the damaged endothelium and secondary release of sustained Xe in the Xe-ELIP. Both have synergic effects: prevention of hematoma expansion and neuroprotection.

Hematoma expansion consists of initial bleeding and re-bleeding. The initial hematoma expansion is via a single vessel that bursts and continues to bleed. It is a short monophasic process, lasting for 1–4 hours and re-bleeding usually occurs within 72 hours^32^. Hematoma expansion strongly influences mortality. Clinical data has demonstrated that nearly two thirds of deaths following SAH were due to the initial hemorrhage, and almost all of these deaths occurred during the first 2 days. Re-bleeding accounted for 22% of the 30-day mortality, whereas delayed arterial vasospasm contributed to only 6% of deaths^33^. The mortality hazard ratio goes up by 5% with every 10% increase in hemorrhagic volume^34^. Rapid repair (i.e., sealing) of microvascular endothelium damage is necessary for cell survival^35^.

We found that ELIP only treatment slightly decreased hemorrhagic volume. A possible hypothesis for the effect of ELIP is that, following SAH, the injury causes vascular or cell membrane disruption, exposing positive charges underneath the membrane^36^. Particles such as ELIP that have negative charges should theoretically be able to attach to the injured blood vessel wall and reseal damaged membranes (Fig. 7b). This hypothesis confirmed by others who have used cell membrane cytoskeleton-targeted immunoliposomes to reseal damaged membranes and ameliorate vascular leakage^37^.

Mechanisms leading to SAH re-bleeding are complicated. Vessel constriction caused by impaired nitric oxide (NO), endothelin, neutriphil, and potassium channels are potential causes^38^. Dilatation of the cerebral arteries in response to ATP-sensitive potassium channel activators reverses vasospasm^39^. Xe affects the opening of the adenosine triphosphate-sensitive potassium (KATP) channels^40^. In addition, Xe interacts with the human immune system by modulating inflammatory cytokines such as TNF-α and IL-6 in monocytes^20,41^. Xe helps sustain release of hypoxia inducible factor-1 alpha (HIF-1α) and other proteins^42–46^ such as heme oxygenase 1 (HO-1)^47^ which are highly related to hemostasis^48^. We found that Xe inhibited neutrophil invasion into the brain (Fig. 6i), supporting our hypothesis that Xe reduces the inflammatory response post SAH.

Evaluation of neuronal cell death by TUNEL staining demonstrated that SAH induced a marked increase in the incidence of TUNEL-positive cells. Neuronal injury was clinically evaluated by behavioral tests. Xe-EILP treatment reduced the number of TUNEL-positive cells to 72 ± 9 (P < 0.01 vs. SAH, Fig. 6f,g) and prevented the impairment of neurological function, while ELIP only treatment did not improve neuronal cell death (284 ± 48, P = 0.96 vs. SAH, Fig. 6f) or neurological functions. This supports our hypothesis that the major neuroprotective effect is caused by Xe, not by the lipids.

Xe neuroprotection has a biphasic mechanism: early stage anti-excitotoxic effect for early stabilization, and long-lasting cytoprotection of the neurovascular unit. The latter is induced by increasing the brain-derived neurotrophic factor (BDNF) and pro-survival proteins such as Bcl-2 that promote cell tolerance to ischemic injury^20^. Our data demonstrated that Xe-solution treatment did not provide any therapeutic effect (TUNEL positive cells = 361 ± 64, P = 0.25 vs. SAH, Fig. 6f) and had impaired neurological function (Fig. 3). This demonstrates that Xe delivery without a carrier may not allow a sufficient local concentration of Xe or later Xe delivery for neuroprotection. ELIP as a carrier can overcome this problem. Additionally, our behavior tests demonstrated that Xe-ELIP greatly improved neurological function following SAH. The secondary release of Xe from the ELIP adhered to endothelial cells may be responsible for this neuroprotection. Thus our two-stage delivery from our Xe carrier has great potential to deliver sufficient therapeutic concentrations to hypoperfused brain tissue to stabilize neurons in the early stages of stroke^21,49^.

As a proof-of-concept, we evaluated the therapeutic effects of Xe-ELIP using a 3-day survival animal model in this study. However, SAH causes prolonged pathologic changes. Within 3 days after onset of SAH, brain tissue goes through a number of pathological process including inflammation. In addition, a second wave of injury occurs at much later time points. In order to take our therapeutic technology forward as a clinical treatment, we are currently investigating an optimized multiple drug delivery strategy for a longer lasting effect as the tissue undergoes late injury and repair.

Therapeutic delivery to the brain following stroke onset is a challenge. Continuing our previous research on the neuroprotective effects of Xe-ELIP for ischemic stroke, this study demonstrates that Xe alleviates early brain injury after SAH by decreasing hematoma, inhibiting neuronal cell death, and improving neurological behavioral deficits. Together, our studies indicate that Xe-ELIP provides therapeutic effects for both ischemic and hemorrhagic stroke. Unlike other neuroprotectants, Xe has unique advantages, including rapid diffusion across the BBB and essentially no side effects. These advantages allow its use as a cytoprotective agent for treatment of both ischemic and hemorrhagic stroke. The two-stage delivery from our Xe carrier has great potential to deliver sufficient therapeutic concentrations to hypoperfused brain tissue in early stages of stroke.

Together, our studies demonstrate the benefit of early Xe delivery through our therapeutic carrier for both SAH and ischemic stroke. This strategy can be given at the time of stroke identification irrespective of stroke type with immediate neuroprotection extending the time window for additional therapeutics, while providing stabilization of the neurovascular unit.

## Methods

### Preparation of Xe-containing Echogenic Liposomes (Xe-ELIP)

Liposomes were composed of 1,2-dipalmitoyl-*sn*-glycero-3-phosphocholine (DPPC; Avanti Polar Lipids, Alabaster, Ala); Egg phosphocholine (Egg-PC; Avanti Polar Lipids); 1,2-dipalmitoyl-*sn-*glycero-3-phosphoethanolamine-N-[methoxy(polyethylene glycol)-2000] (PEG2000 PE) 1,2-dipalmitoyl-*sn*-glycero-3-phospho-(1′-*rac*-glycerol) (DPPG), and cholesterol (Sigma, St Louis, Mo) at a molar ratio of 43:28:6:8:15. This lipid composition has a similar acoustic response^50^ with higher stability than our early formulations^51^. FITC-labeled ELIP were prepared by adding 0.2% FITC-labeled 1,2-dioleoyl-*sn*-glycero-3-phosphoethanolamine.

Xe-ELIP were prepared by the previously described pressurization-freeze method^15,24^. Brifely, lipid mixtures (5 mg total weight) were dried in a 2-ml (15 × 45 mm) rubber septa screw-cap borosilicate glass vials (12 × 32 mm) by evaporation under argon in a 50 °C water bath with constant rotation. The lipid film was then placed under high vacuum (<100 mtorr) for 4–6 hours for complete removal of solvent. The dried lipid film was hydrated with 500 μl 0.32 M mannitol. Xe gas (6 ml) was introduced into the hydrated lipid dispersion through the septum. The pressurized-gas/liposome dispersion was incubated for 30 minutes at room temperature, and then frozen at −80 °C. The pressure was released by unscrewing the caps immediately after their removal from freezer. The depressured frozen liposomes were then thawed with a loose cap at room temperature.

### Intravascular Ultrasound Imaging of Xe Bubbles in ELIP

Xe-ELIP in a 5-ml vial were imaged using a 15-MHz intravascular ultrasound (IVUS) catheter (Boston Scientific Scimed Inc, Maple Grove, MN). IVUS images of Xe-ELIP were acquired and digitized to 640 × 480-pixel spatial resolution (approximately 0.045 mm/pixel) and 8-bit (256 level) amplitude resolution. Assessment of echogenicity (apparent brightness) of Xe-ELIP in the IVUS images was performed using Image Pro-Plus Software (Media Cybernetics, Silver Spring, MD). Data were reported as mean grayscale values^24,52^.

### Transmission Electronic Microscopy Imaging

Transmission electron microscopy (TEM) was performed to determine the bilayer structure of the gas-containing ELIP. A small amount (10 μl) of hydrated gas-containing ELIP were placed onto a grid, and the background was stained using a negative stain, Uranyl Acetate. The outer portion of each vesicle is shown in black whilst the inner area is white. As the space in the TEM chamber was in a vacuum state, gas bubbles (undissolved gas) in the liposomal structure are eliminated. Therefore, the remaining images allowed evaluation of the dissolved gas within the bilayer.

### Determination of Xe Concentration in Xe-ELIP

Xe concentration was measured by GC-MS (HP 6890-HP 5973A) with a G1512 controller, GC injector (Tower and Tray), and Edwards E2M2 rough pump. Gases were separated with a Restek 30 m, 0.32 mm ID Rt-Msieve 5A fused silica PLOT molecular sieve column interfaced with the MS through a 2.5-m particle trap. Volumes of pure Xe gas were removed from a small compressed gas cylinder (Matheson), transferred to 2-ml glass vials, and sealed with Teflon-lined screw-top caps to determine vol/vol percent standards. A Xe standard curve (AUC vs. %Xe) was prepared and linear regression performed to calculate Xe% in the sample headspace. This was converted to Xe mass in micrograms based on a molar gas volume of 25.5 liters at 37 °C (which yields mM) at an atomic weight of 132. Micrograms Xe per mg lipid was determined by dividing this value by the sample volume. To measure Xe concentration in Xe-ELIP, 100-µl aliquots of Xe-ELIP samples (10 mg lipids/ml) were transferred to a fresh sampling vial, and 5 µl was sampled from the headspace and manually injected into the GC for autosampling. The peak areas (AUC) were determined in the auto-integration mode.

### Determination of ELIP Adhesion to Damaged Endothelial Cells

ELIP adhesion to damaged endothelial cells was investigated *in vitro* using cultured human umbilical vein endothelial cells (HUVEC). HUVEC were grown in 75-cm^2^ flasks containing a medium of Dulbecco modified Eagle medium (DMEM) (PromoCell, Heidelberg, Germany) with supplement (5% fetal bovine serum (FBS), 100 U/ml penicillin, and 100 mg/ml streptomycin) at 37 °C under 5% CO_2_ and 95% air. Cells obtained between passages 4 and 6 were used. Twenty-four hours before treatment, HUVEC were seeded in a 24-well plates and treated with TNF-α (50 ng/ml) to induce endothelial cell damage. FITC-labeled ELIP were added to normal HUVEC and TNF-α-treated HUVEC. Three hours after treatment, adhesion of FITC-ELIP was observed by fluorescence microscopy.

### Subarachnoid Hemorrhage Model and Experimental Groups

All methods were carried out in accordance with relevant guidelines and regulations, and all experimental protocols of animal studies were approved by the Animal Welfare Committee at The University of Texas Health Science Center at Houston. Sprague-Dawley male rats (Harlan Laboratories Inc., Indianapolis, IN) weighing 320–360 g were kept on a 12-hour light/dark cycle under controlled temperature conditions (22 ± 2 °C) and fed standard foods and water. Animals were randomly divided into five groups: sham control (Sham), SAH with no treatment (SAH), SAH treated with Xe-ELIP (Xe-ELIP), SAH treated with empty ELIP (ELIP), and SAH treated with Xe-saturated solution (Xe solution).

Prior to surgical intervention, all animals were fasted for 72 hours with free access to water. Animals were anesthetized using mechanical ventilation with 2% isoflurane in oxygen (2/1) and received a subcutaneous injection of marcaine (2 mg/kg) to provide topical analgesia before incision. During the operation, a temperature-controlled heating pad was used to maintain the rectal temperature at 37.5 °C. SAH was induced by endovascular perforation near the left internal carotid artery (ICA) bifurcation using a monofilament 4–0 nylon suture^53^. After exposing the left carotid artery and its branches, the external carotid artery (ECA) was transected distally and fashioned into a stump. The suture was advanced into ICA through the common carotid artery (CCA) bifurcation and further advanced into the intracranial ICA until resistance was felt approximately 18–20 mm from the CCA bifurcation. Further pushing of the suture for approximately 3 mm perforated the Circle of Willis. The suture was withdrawn after 15 seconds and ICA was reperfused. Sham-operated animals underwent the same procedure except that the suture was removed without puncture when resistance was felt.

### Xe-ELIP Administration

At 30 minutes after onset of SAH, Xe-ELIP (600 μl per animal, 10 mg lipid/ml), ELIP (600 μl per animal, 10 mg lipid/ml), or Xe solution (600 μl per animal) were infused into the tail vein using a 21 gauge needle over a period of 15 minutes. An ultrasound probe (diameter 1.2 cm) was placed 5 mm above the left ICA (Fig. 7a) and 1-MHz continuous wave ultrasound (a peak-to-peak pressure amplitude of 0.18 MPa) was applied to trigger Xe release from Xe-ELIP during the liposomal infusion. Phosphate-buffered saline (PBS) solution was filled between the artery and ultrasound probe to ensure adequate acoustic coupling.

### Regional Blood Flow Perfusion Monitor

Cerebral blood flow was monitored in the middle cerebral artery (MCA) circulation region (2 mm lateral and 2 mm posterior to the bregma) before and after MCA perforation using a PR407–1 straight-needle laser Doppler flow meter probe (Perimed, Sweden) connected to a standard laser Doppler monitor (PF5010 LDPM unit and PF5001 main unit; Perimed, Sweden). SAH onset was confirmed by the interruption of blood flow (50–70%) in each animal, and by autopsy after euthanization. All animals were allowed to recover from anesthesia 2 hours after SAH onset and housed individually.

### Neurological Evaluation

Neurological scores were determined using an 18-point scoring system to assess sensorimotor deficits^54^ before euthanization in a blinded fashion at 72 hours. There were six catalogs in this assessment – spontaneous activity, spontaneous movements of limbs, forelimbs outstretching, climbing a wall of a wire cage, axillary touch response, and response to vibrissae touch. The worst score was 0 and the best was 3 for each subtest, and the total score was summarized from all of the subtests (shown in Table 1).

### Locomotion Behavioral Tests

Beam walking and grid walking tests were performed to evaluate motor function following SAH. The beam walking test (0–6 grading) was used to assess the ability to walk across and maintain balance on a beam (2.5 × 2.5 × 80 cm). The response scores were assigned as follows: score 0 – traversed the beam with no foot slip; score 1 – traversed with grasping of the lateral side of the beam; score 2 – showed difficulty crawling across the beam but able to traverse; score 3 – required more than 10 seconds to traverse the beam due to difficulty in walking; score 4 – unable to traverse the beam; score 5 – unable to move the body or any limb on the beam; score 6 – unable to stay on the beam for more than 10 seconds. Grid walking test was performed by placing the animal on a stainless steel grid floor (20 cm × 40 cm with a mesh size of 2 cm × 2 cm). The total number of steps was counted up to the maximum of 50 steps. The number of foot fault errors, i.e., misplacement of a forelimb or hindlimb falling through the grid was recorded.

### SAH Grading

Grading of the severity of SAH was evaluated at 72 hours after surgery in all animals^54^. After removing the brain, a picture of the basal cistern was taken. The basal cistern area was divided into 6 subsections including the left and right frontal, left and right temporal, and upper and lower brain stem. Depending on the prevalence of subarachnoid blood clots, every segment was given a grade from 0 to 3: Grade 0 - no subarachnoid blood clot; Grade 1 - minimal subarachnoid blood; Grade 2 - moderate blood clot with recognizable arteries; Grade 3 - blood clot obliterating all arteries within the segment. The total score (maximum: 18) was calculated as the sum of the scores from the 6 subsections.

### Spectrophotometric Assay Hemorrhagic Volume

To further confirm the effect of Xe-ELIP on hematoma development, hemorrhagic volume was quantitated using colorimetric method^55^. Animals were randomly divided into three groups: sham control (Sham), SAH no treatment (SAH), and SAH treated with Xe-ELIP (Xe-ELIP). At 2 hours after SAH onset, animals were euthanized and the brains were harvested. A standard curve was obtained by adding incremental volumes of homologous blood (0, 2, 4, 8, 16, 32, 64, 128, 256 and 512 μl) to the brain tissue obtained from perfused normal rats. Samples were homogenized after adding PBS until reaching a total volume of 3 ml, and centrifuged at 13,000 rpm for 30 minutes. Drabkin’s reagent (1.6 mL, Sigma) was added to 0.4-mL aliquots of the supernantes to incubate for 15 minutes at room temperature. Absorbance at 540 nm was measured using a spectrophotometer (Spectronix 3000, Milton-Roy, Rochester, NY, U.S.A.). Data were presented in terms of hemorrhage volume (μl).

### *In Situ* Labeling of DNA Fragmentation

Animals were sacrificed 72 hours after SAH onset. Freshly frozen brains were coronally cut into 5 μm thick sections were subjects to TUNEL staining to assess DNA fragmentation in degenerating neurons. The slides were fixed in 4% paraformaldehyde for 20 minutes at room temperature and permeabilization was performed using 0.1% Triton X-100 and 0.1% sodium citrate for 2 minutes at 4 °C. Each slide was incubated with 50 μl TUNEL reaction mixture (Roche Diagnostics GmbH, Mannheim, Germany) at 37 °C for 1 hour. The slides were mounted with prolonged anti-fade mountant with DAPI (Invitrogen, Oregon, USA), and covered using a coverslip. Images were viewed under a Nikon ECLIPSE Ti fluorescence microscope and acquired using a CoolSNAP photomatrics camera. TUNEL-positive cells (green fluorescence) were quantitatively determined at 100 × magnification in 3 fields close to the subcortex area using diagnostic software (NIS-Elements BR 3.2)^31^.

### Double Immunofluorescence Staining

To confirm severity of neuronal damage, freshly frozen brains from the Sham, SAH and Xe-ELIP groups were coronally cut into 5-μm thick sections and subjected to TUNEL staining and caspase 3 staining (Santa Cruz Biotechnology; diluted 1:50). To counterstain the neurons, astrocytes, and endothelial cells, sections were incubated with an antibody against a neuron-specific nuclear protein (NeuN; Millipore, Temecula, CA, USA, diluted 1:200).

To investigate the invasion of neutrophils into the brain, frozen brain tissue was stained using neutrophil elastase (NeuN; abcam, Temecula, CA, USA, diluted 1:200). The blood vessl was demonstrated by staining α-actin (Santa Cruz Biotechnology; diluted 1:1000), and colocalized with neutrophils. Alexa Fluor 488 Goat Anti-Rabbit IgG and Alexa Fluor 555 Goat Anti-Mouse IgG (Life technologies, Grand Island, NY, USA, diluted 1:1000) were used as secondary antibodies. Staining data were examined by fluorescent microscopy (Nikon, Ti-U, Dusseldorf, Germany).

### Statistical Analysis

All data were presented as mean and standard error of the mean (SEM). Statistical significance was examined using the Kruskal-Wallis ANOVA of ranks and median test, followed by post hoc multiple comparisons of mean ranks for all groups for each comparison. A value of P < 0.05 was considered significant. SPSS 13.0 (SPSS Inc, Chicago, IL) was used for all statistical analyses.

### Data Availability

The datasets generated during and/or analysed during the current study are available from the corresponding author on reasonable request.
